# Supplementary material for: Am I truly monolingual? Exploring foreign language experiences in monolinguals
Source: PLoS One. 2022 Mar 21;17(3):e0265563. doi: 10.1371/journal.pone.0265563 (PMC8936441; doi:10.1371/journal.pone.0265563)
Supplement: S1 Appendix — (DOCX) [file pone.0265563.s001.docx]

**S1 Appendix. Pre-screening filters (Prolific).**

1. What is your country of birth? (Select from the dropdown list)
2. In what country do you currently reside? (Select from the dropdown list)
3. Are you an English-speaking monolingual, that is, are you fluent only in English? Or are you also fluent in any other language(s)?

I only know English.

I know one other language in addition to English.

I know 2 or more languages in addition to English.

N/A or rather not say.

1. Apart from your native language, do you speak any other language fluently?

none just my native language.

native language + one other language.

native language + two other languages.

native language + three other languages.

1. Were you raised monolingual?

I was raised with my native language only.

I was raised with two or more languages.

1. Do you have any language related disorders?

reading difficulty.

writing difficulty

other language related disorder.

none.

not applicable.

Participants were invited to the study if they reported United Kingdom as their country of birth and current country of residence, and if they answered “I only know English”, “none just my native language”, “I was raised with my native language only”, and “none” to questions 3, 4, 5, and 6. In addition, participants were invited to the study only if they had at least an 80% approval rate from Prolific, had completed at least 10 studies in Prolific, and had not participated in any previous related study.
