# Supplementary material for: Am I truly monolingual? Exploring foreign language experiences in monolinguals
Source: PLoS One. 2022 Mar 21;17(3):e0265563. doi: 10.1371/journal.pone.0265563 (PMC8936441; doi:10.1371/journal.pone.0265563)
Supplement: S2 Appendix — (DOCX) [file pone.0265563.s002.docx]

**S2 Appendix. Complete survey.**

**Section 1: Learning languages.**

- Have you ever learned another language apart from your native language, including a dialect or jargon (in or outside school)? Yes  No
  - *If applicable:* Please, indicate the name of the language(s)/dialect(s) and for how long (in years or months) you learned each of them. Indicate if the reported time corresponds to years or months. If you learned more than three languages/dialects apart from your native language, report the three that you learned for a longer period. Example: Language/dialect 1: French, 2 years. Language/dialect 2: Spanish, 6 months

|  | **Name of the language/dialect**  *Write the name of the language/dialect* | **Time learning**  *Write the number (e.g., 6)* | **Years or months?**  *Select from the dropdown list* |
| --- | --- | --- | --- |
| Language/dialect 1 |  |  | Months  Years |
| Language/dialect 2 |  |  | Months  Years |
| Language/dialect 3 |  |  | Months  Years |

**Section 1A: English proficiency.**

- Compare yourself with a highly proficient speaker and rate your proficiency in English. Base your ratings on a scale from 0 (no proficiency) to 10 (native’s speaker knowledge) according to the following skills: reading, listening, writing and speaking.
  - Reading: 0 (no knowledge) – 1 – 2 – 3 – 4 – 5 – 6 – 7 – 8 – 9 – 10 (native speaker)
  - Listening: 0 (no knowledge) – 1 – 2 – 3 – 4 – 5 – 6 – 7 – 8 – 9 – 10 (native speaker)
  - Writing: 0 (no knowledge) – 1 – 2 – 3 – 4 – 5 – 6 – 7 – 8 – 9 – 10 (native speaker)
  - Speaking: 0 (no knowledge) – 1 – 2 – 3 – 4 – 5 – 6 – 7 – 8 – 9 – 10 (native speaker)
- At what age did you start learning English? (if from birth, write 0): ______
- At what age did you start using English (when you were able to take part in a simple conversation in that language)? ______

**Section 1B: Proficiency in other languages/dialects** *(if applicable).*

- Compare yourself with a highly proficient speaker and rate your proficiency in __________ (*name* *language/dialect 1)*. Base your ratings on a scale from 0 (no proficiency) to 10 (native’s speaker knowledge) according to the following skills: reading, listening, writing and speaking.
  - - Reading: 0 (no knowledge) – 1 – 2 – 3 – 4 – 5 – 6 – 7 – 8 – 9 – 10 (native speaker)
    - Listening: 0 (no knowledge) – 1 – 2 – 3 – 4 – 5 – 6 – 7 – 8 – 9 – 10 (native speaker)
    - Writing: 0 (no knowledge) – 1 – 2 – 3 – 4 – 5 – 6 – 7 – 8 – 9 – 10 (native speaker)
    - Speaking: 0 (no knowledge) – 1 – 2 – 3 – 4 – 5 – 6 – 7 – 8 – 9 – 10 (native speaker)
    - At what age did you start learning __________ (*name* *language/dialect 1)*? (if from birth, write 0): ______
    - Compare yourself with a highly proficient speaker and rate your proficiency in __________ (*name language/dialect 2)*. Base your ratings on a scale from 0 (no proficiency) to 10 (native’s speaker knowledge) according to the following skills: reading, listening, writing and speaking.
    - Reading: 0 (no knowledge) – 1 – 2 – 3 – 4 – 5 – 6 – 7 – 8 – 9 – 10 (native speaker)
    - Listening: 0 (no knowledge) – 1 – 2 – 3 – 4 – 5 – 6 – 7 – 8 – 9 – 10 (native speaker)
    - Writing: 0 (no knowledge) – 1 – 2 – 3 – 4 – 5 – 6 – 7 – 8 – 9 – 10 (native speaker)
    - Speaking: 0 (no knowledge) – 1 – 2 – 3 – 4 – 5 – 6 – 7 – 8 – 9 – 10 (native speaker)
- At what age did you start learning __________ (*name* *language/dialect 2)*? (if from birth, write 0): ______
- Compare yourself with a highly proficient speaker and rate your proficiency in __________ (*name* *language/dialect 3)*. Base your ratings on a scale from 0 (no proficiency) to 10 (native’s speaker knowledge) according to the following skills: reading, listening, writing and speaking.
  - - Reading: 0 (no knowledge) – 1 – 2 – 3 – 4 – 5 – 6 – 7 – 8 – 9 – 10 (native speaker)
    - Listening: 0 (no knowledge) – 1 – 2 – 3 – 4 – 5 – 6 – 7 – 8 – 9 – 10 (native speaker)
    - Writing: 0 (no knowledge) – 1 – 2 – 3 – 4 – 5 – 6 – 7 – 8 – 9 – 10 (native speaker)
    - Speaking: 0 (no knowledge) – 1 – 2 – 3 – 4 – 5 – 6 – 7 – 8 – 9 – 10 (native speaker)
- At what age did you start learning __________ (*name* *language/dialect 3)*? (if from birth, write 0): ______
  - *If applicable:* Have you ever used any of the language(s)/dialect(s) that you learned for any reason? Yes  No

**Section 2: Living abroad.**

- Have you ever lived in a country in which a language or dialect other than your native language was spoken as a majority language? Yes  No
  - *If applicable:* Please, indicate the name of the country, the length of your stay (in months or years) and the language(s)/dialect(s) spoken. Indicate if the reported length corresponds to months or years. If you lived in more than four countries, report the four in which you lived longer. Examples: France, 1 year, French. Italy, 6 months, Italian.

|  | **Country**  *Write the name of the country* | **Length**  *Write the number (e.g., 3)* | **Years or months?**  *Select from the dropdown list* | **Languages/dialects spoken**  *Write the name of the language/dialect* |
| --- | --- | --- | --- | --- |
| Country 1 |  |  | Months  Years |  |
| Country 2 |  |  | Months  Years |  |
| Country 3 |  |  | Months  Years |  |
| Country 4 |  |  | Months  Years |  |

- - *If applicable:* While living abroad, did you use any other language(s)/dialect(s) apart from your native language to communicate? Yes  No
  - *If applicable:* Are the language(s) or dialect(s) from these countries currently present in your environment? Yes  No
  - *If applicable:* Do you currently use any of the language(s)/dialect(s) of those countries for any reason? Yes  No

**Section 3: Exposure to other languages in the UK.**

- While living in the UK, have you ever been exposed to any other language(s)/dialect(s) apart from your native language? Yes  No
- *If applicable:* Please, indicate the name of the language(s)/dialect(s) and for how long (in years or months) you were or have been exposed to them: Indicate if the reported length corresponds to years or months If you have been exposed to more than 3 languages/dialects, report the 3 languages/dialects to which you have been exposed for a longer period of time.

|  | **Name of the language/dialect**  *Write the name of the language/dialect* | **Time exposed**  *Write the number (e.g., 2)* | **Years or months?**  *Select from the dropdown list* |
| --- | --- | --- | --- |
| Language/dialect 1 |  |  | Months  Years |
| Language/dialect 2 |  |  | Months  Years |
| Language/dialect 3 |  |  | Months  Years |

- *If applicable:* Have you ever used any of the language(s)/dialect(s) to which you have been exposed in the UK for any reason? Yes  No

**Section 4. Passive use of languages.**

- Now we would like to learn how you passively use your languages on a typical day. With the COVID-19 crisis, your typical day might have changed. For example, it might be possible that right now you are working or learning from home. All these changes might have also altered how you use languages when you watch TV, listen to music or radio, browse on the internet, etc. Has the COVID-19 crisis modified your passive use of languages?

Yes, my passive use of languages has changed since the COVID-19 crisis.

No, my passive use of languages has not changed since the COVID-19 crisis.

- **If yes:** Think about your passive use of languages (i.e., watching movies or series, browsing on the internet, listening to music or radio) before and during the COVID-19 crisis. Estimate how much you used/use each language passively. Remember to write the name of each language. Give an answer for each language as a percentage. Remember that the sum of the percentages should add up to 100% (e.g., English 60%, Spanish 40%).
  - Before the COVID-19 crisis:
    - Name language 1: _________, ___ %
    - Name language 2: _________, ___ %
    - Name language 3: _________, ___ %
    - Name language 4: _________, ___ %
  - During the COVID-19 crisis:
    - Name language 1: _________, ___ %
    - Name language 2: _________, ___ %
    - Name language 3: _________, ___ %
    - Name language 4: _________, ___ %
- **If no:** Think about your passive use of languages (i.e., watching movies or series, browsing on the internet, listening to music or radio). Estimate how much you use each language passively. Remember to write the name of each language. Give an answer for each language as a percentage. Remember that the sum of the percentages should add up to 100% (e.g., English 60%, Spanish 40%).
  - - Name language 1: _________, ___ %
    - Name language 2: _________, ___ %
    - Name language 3: _________, ___ %
    - Name language 4: _________, ___ %
