# Supplementary material for: Am I truly monolingual? Exploring foreign language experiences in monolinguals
Source: PLoS One. 2022 Mar 21;17(3):e0265563. doi: 10.1371/journal.pone.0265563 (PMC8936441; doi:10.1371/journal.pone.0265563)
Supplement: S1 Table — Number of participants is shown between brackets. (DOCX) [file pone.0265563.s003.docx]

**S1 Table. List of foreign languages/dialects/types of jargon learned by participants. Number of participants is shown between brackets.**

| List of foreign languages/dialects/jargon | | | |
| --- | --- | --- | --- |
| Afrikaans (2)  Ancient Greek (1)  Arabic (3)  Back slang (1)  British Sign Language (3)  Bulgarian (1)  Cantonese (1)  Czech (2)  Danish (1)  Dari (1)  Doric (2)  Dutch (1) | Finnish (1)  French (678)  French Creole (1)  Gaelic (4)  German (333)  Greek (4)  Hebrew (3)  Hindi (1)  Hungarian (2)  Irish (5)  Italian (35)  Japanese (18)  Korean (3) | Latin (42)  Luganda (1)  Malay (2)  Mancunian (1)  Mandarin (6)  Mandinka (1)  Norwegian (1)  Polish (4)  Portuguese (4)  Romanian (2)  Russian (7)  Scots (3)  Sign Language (3)^a^ | Sindarin (1)^b^  Sinitic languages (1)^c^  Slovak (1)  Spanish (235)  Swahili (1)  Swedish (1)  Tagalog (1)  Tamil (1)  Thai (4)  Turkish (3)  Vietnamese (1)  Welsh (23) |

^a^ Participants did not specify which Sign Language.

^b^ Sindarin is an artificial language created by J. R. R. Tolkien.

^c^ This participant did not specify which Sinitic language (i.e., reported “Chinese”).
