# Supplementary material for: Am I truly monolingual? Exploring foreign language experiences in monolinguals
Source: PLoS One. 2022 Mar 21;17(3):e0265563. doi: 10.1371/journal.pone.0265563 (PMC8936441; doi:10.1371/journal.pone.0265563)
Supplement: S2 Table — (DOCX) [file pone.0265563.s004.docx]

**S2 Table. List of foreign languages/dialects to which participants have been exposed in the UK.**

| List of foreign languages/dialects | | |
| --- | --- | --- |
| Afrikaans (2)  Arabic (17)  Bengali (2)  Bosnian (1)  British Sign Language (1)  Bulgarian (1)  Cockney (1)  Creole (1)  Czech (3)  Danish (1)  Devonian English (1)  Doric (2)  Dutch (2)  East midlands English (1)  Farsi (1)  Fijian (1)  Filipino (2)  Finnish (1)  Flemish (2)  French (71)  French Creole (1)  Gaelic (9)  German (37) | Greek (7)  Gujarati (4)  Hindi (14)  Hungarian (3)  Irish (2)  Italian (13)  Japanese (4)  Languages spoken in India (11)^a^  Languages spoken in South Africa (1)^a^  Languages spoken in Sri Lanka (1)^a^  Languages spoken in the African continent (2)^a^  Latvian (1)  Lithuanian (2)  Malay (1)  Mandarin (3)  Mandinka (1)  Mauritian creole (1)  Pakistani (3)  Patois (1) | Pidgin (1)  Polish (74)  Portuguese (11)  Punjabi (11)  Romanian (7)  Russian (7)  Scots (2)  Scottish (2)  Sinitic languages (7)^b^  Slovak (1)  Spanish (58)  Tagalog (1)  Telugu (1)  Tamil (1)  Thai (5)  Turkish (3)  Urdu (26)  Welsh (37)  West midlands English (1)  Yoruba (2) |

^a^ These participants mislabelled the language (e.g., “South African”, “Indian”, “Sri Lankan”).

^b^ These participants did not specify which Sinitic language (i.e., “Chinese”).
