# Supplementary material for: Am I truly monolingual? Exploring foreign language experiences in monolinguals
Source: PLoS One. 2022 Mar 21;17(3):e0265563. doi: 10.1371/journal.pone.0265563 (PMC8936441; doi:10.1371/journal.pone.0265563)
Supplement: S3 Table — Number of participants is shown between brackets. (DOCX) [file pone.0265563.s005.docx]

**S3 Table. List of languages/dialects passively used by participants. Number of participants is shown between brackets.**

| Languages passively used by participants | | |
| --- | --- | --- |
| Bengali (1)  British Sign Language (1)  Bulgarian (1)  Czech (1)  Danish (2)  Danish/Swedish (1)^a^  Dutch (2)  English (941)  Finnish (1)  French (104)  Gaelic (4)  German (47)  Greek (2)  Hindi (2) | Irish (2)  Italian (15)  Japanese (21)  Korean (8)  Languages spoken in India (1)^b^  Maltese (1)  Mandarin (1)  Norwegian (2)  Polish (6)  Portuguese (3)  Punjabi (1)  Romanian (2)  Russian (8)  Scottish (1) | Scots (3)  Sindarin (1)^c^  Sinitic languages (2)^d^  Slovak (1)  Spanish (104)  Swedish (3)  Tamil (1)  Thai (2)  Turkish (2)  Welsh (9) |

^a^ One participant reported the passive use of Danish and Swedish together.

^b^ One participant mislabelled the language (i.e., they reported “Indian”).

^c^ Sindarin is an artificial language created by J. R. R. Tolkien.

^d^ These participants did not specify which Sinitic language (i.e., they reported “Chinese”).
