# Supplementary material for: Am I truly monolingual? Exploring foreign language experiences in monolinguals
Source: PLoS One. 2022 Mar 21;17(3):e0265563. doi: 10.1371/journal.pone.0265563 (PMC8936441; doi:10.1371/journal.pone.0265563)
Supplement: S4 Table — Number of participants is shown between brackets. (DOCX) [file pone.0265563.s006.docx]

**S4 Table. Non-English-speaking countries reported as temporary countries of residence. Number of participants is shown between brackets.**

| List of non-English-speaking countries | | | |
| --- | --- | --- | --- |
| Afghanistan (1)  Austria (2)  Belgium (1)  Bulgaria (1)  Brazil (2)  Cambodia (1)  Canada (1)  Costa Rica (1)  Cyprus (3)  Czech Republic (1)  Denmark (1)  Egypt (1) | France (13)  Germany (8)  Greece (4)  Guatemala (1)  Hong Kong (4)^a^  Iceland (1)  India (5)  Indonesia (1)  Ireland (1)  Israel (1)  Italy (2)  Japan (5) | Kenya (1)  Laos (1)  Malaysia (2)  Malta (2)  Mexico (1)  Nicaragua (1)  Peru (1)  Philippines (1)  Qatar (1)  Saudi Arabia (1)  Singapore (2)  South Africa (3) | South Korea (1)  Spain (17)  Sweden (1)  Switzerland (2)  Taiwan (1)  Thailand (5)  the Netherlands (5)  Tunisia (1)  Turkey (1)  Vietnam (2)  Wales (4)  Yemen (1) |

^a^ Hong Kong is a Special Administrative Region controlled by the Republic of China, with a prevalence of Cantonese instead of Mandarin Chinese (the majority language in China).
