# Supplementary material for: Am I truly monolingual? Exploring foreign language experiences in monolinguals
Source: PLoS One. 2022 Mar 21;17(3):e0265563. doi: 10.1371/journal.pone.0265563 (PMC8936441; doi:10.1371/journal.pone.0265563)
Supplement: Attachment — Submitted filename: Response to Reviewers.docx [file pone.0265563.s008.docx]

**Response to Reviewers**

**Thank you for submitting your manuscript to PLOS ONE. After careful consideration, we feel that it has merit but does not fully meet PLOS ONE’s publication criteria as it currently stands. Therefore, we invite you to submit a revised version of the manuscript that addresses the points raised during the review process.**

We appreciate the comments and suggestions provided by the Reviewer. All points have been addressed in the revised version of the manuscript. In addition, in the revised version, the reference number 54 has been updated from a preprint to a published article (Voits et al., 2022; page 23, line 504 and page 32, line 760 of the clean version). We have also included three new references in the revised version (numbers 27, 28, 29). The in-text citations for these references are on page 4, line 90 (Byers-Heinlein et al., 2017) and line 92 (Oh et al., 2020; Tobin et al., 2017) of the clean version.

**Reviewer #1:**

**This is a revised version of a previously submitted manuscript examining variability in language experience in monolingual (native English) speakers. The authors have done a good job of addressing the comments from the previous version of the manuscript, and the current version is certainly improved. In reading the manuscript again, I have a couple further comments which I would like to see addressed but am otherwise happy to recommend for publication.**

**Comments:**

1. **In the ‘Insights for Future Research’ section, it would be useful if the authors could expand on their calls to include separate questions passive exposure in the current bilingual questionnaires. If these are included as a separate section or set of questions, would these questions directly overlap directly with questions pertaining to active use? That is, would the same scenarios be examined, just with passive use?**

If we understand correctly, the Reviewer is asking us to clarify the difference between passive exposure and passive use. Our suggestion to include specific questions on passive exposure in addition to passive use reflects our interest in obtaining information on whether participants frequently encounter foreign languages without interacting with them or without even paying attention to them (e.g., people who have foreigners as neighbours and hear them talking in their native language in the vicinity; people who have foreign co-workers who speak in their native language at work). On the other hand, questions on passive use would provide information on whether participants intentionally engage in activities that involve reading or listening in a foreign language. Examples of passive use would be reading a supermarket leaflet in a foreign language or listening to radio advertisements in a foreign language. As passive exposure does not require interacting with or using a language, it is possible to be exposed to foreign languages whilst not passively (or actively) using them.

Including separate questions for passive exposure and passive use would provide a more detailed picture of the languages that a participant encounters. In addition, in the case of monolinguals – our target population – passive exposure has been linked to differences in linguistic processing (Bice & Kroll, 2019; Kurkela et al., 2019). Therefore, it is crucial to understand whether monolinguals are passively exposed to foreign languages, as this exposure might have influenced their cognitive processes.

Following the Reviewer’s suggestion and to ensure that both terms are correctly understood and differentiated, we have clarified the difference between passive exposure and passive use the first time these two terms are presented together (page 6, lines 130-136 of the clean version), and we have made an additional clarification in the section “Insights for future research” (page 25, lines 551-566 of the clean version).

In addition, we agree with the Reviewer that participants might feel an overlap between passive exposure and passive use if the definition is not clear for them. Thus, in addition to defining both terms in the questionnaire, we also propose the inclusion of open-ended questions in which participants could specify in which situations they are passively exposed to foreign languages and in which situations they passively use foreign languages (page 25, lines 566-570 of the clean version)

2. **On a similar note, I am not sure I agree with the authors calls to removing terms like ‘speak’ or ‘know’ outright from the existing questionnaires in detailing language use, as opposed to adding ‘learned’ to this list. It would be useful if the authors could clarify this a bit further.**

We agree with the Reviewer that completely removing the terms “speak”, “know”, or “understand” is not necessary, and that the word “learn” could be added instead. The inclusion of all these different terms would allow monolinguals and bilinguals to report all their languages without restricting their responses to the languages they know/speak/understand or to the languages they have learned. In addition, including all terms would facilitate using the same survey for monolinguals and bilinguals. We have made some modifications to the revised version of the manuscript to propose the inclusion of all these terms in the questionnaires (page 24, lines 524-535).

**Minor comments:**

1. **Table 3- the country column is a little confusingly worded to me. I would recommend changing this for clarity to “1 Foreign country, 2 foreign countries…” etc. The same applies to Table 2. I would reword these as “1 foreign language, 2 foreign languages…” etc.**

Thank you very much for this comment. We have carefully considered your suggestion, but we think that changing the labels to “1 Foreign country, 2 Foreign countries…” could be misleading for future readers. The reason for this is that each row does not exclusively include participants who have lived in only one (or two, or three) foreign countries. In other words, data included in “Foreign country 1” contains all participants who have lived in *at least* one foreign country; data included in “Foreign Country 2” includes all participants who have lived in at least two foreign countries, and so on. In our study, 89 participants had lived at least in one foreign country; 22 had lived in at least two foreign countries; six had lived in at least three foreign countries; and three had lived in four foreign countries. For purposes of clarity, we have included this information as a note below Table 3 (page 17, line 368-374 of the clean version).

The same logic applies to Table 2: The “Foreign Language 1” row includes all participants who had been exposed to *at least* one foreign language; the “Foreign Language 2” row includes all participants who had been exposed to at least two foreign languages, and so on. In this case, a total of 376 participants had been exposed to at least one foreign language; 89 participants had been exposed to at least two foreign languages; and 29 participants had been exposed to three foreign languages. As with Table 3, we have included this information as a note below Table 2 (page 15, lines 326-331 of the clean version). In addition, we have provided some information on page 11 (lines 257-261 of the clean version) on how the countries and languages were categorized. We hope that these changes facilitate the understanding of the table and our data.
